# Oxygen inhalation improves postoperative survival in ketamine-xylazine anaesthetised rats: An observational study

**DOI:** 10.1371/journal.pone.0226430

**Published:** 2019-12-13

**Authors:** Mare Mechelinck, Carolin Kupp, Johanne C. Krüger, Moriz A. Habigt, Marius J. Helmedag, René H. Tolba, Rolf Rossaint, Marc Hein

**Affiliations:** 1 Department of Anaesthesiology, Uniklinik RWTH Aachen, Aachen, Germany; 2 Institute for Laboratory Animal Science and Experimental Surgery, Uniklinik RWTH Aachen, Aachen, Germany; 3 Department of General, Visceral and Transplantation Surgery, Uniklinik RWTH Aachen, Aachen, Germany; Boston University, UNITED STATES

## Abstract

**Objective:**

A simple but reliable and safe anaesthetic procedure is required for surgical interventions in small rodents. Combined ketamine and xylazine injections are often used in rats for less invasive surgery, possibly with spontaneous breathing and without airway management. However, there are important pitfalls to be avoided by special precautions and monitoring, as shown subsequently.

**Study design:**

Observational study.

**Animals:**

Twenty-four anaesthetic procedures for bile duct ligation, sham operation or carotid artery dilatation in 20 male Sprague-Dawley rats, preoperatively weighing between 440 and 550 g.

**Methods:**

Intolerable high mortality rates occurred in the first 7 postoperative days while establishing a new experimental model in rats using ketamine-xylazine anaesthesia. Rats were spontaneously breathing ambient air during the first 12 surgeries without airway management. An observed high mortality rate in these animals led to a change in the trial protocol: the insufflation of 2 litres of oxygen per minute via nose cone during the following 12 rat surgeries. Retrospective comparison of the outcome (without oxygen vs. with oxygen insufflation) was conducted.

**Results:**

The perioperative mortality rate could be significantly reduced from 58% (7/12) to 17% (2/12) (*p* = 0.036) by oxygen insufflation via nose cone. Significantly different levels of intraoperative oxygen saturation (SpO_2_; 89 ± 4% [without oxygen] vs. 97 ± 0.5% [with oxygen], *p* < 0.0001), but no significant differences in heart rate (HR; 267 ± 7 beats minute^–1^ [bpm] [without oxygen] vs. 266 ± 6 bpm [with oxygen], *p* = 0.955) were observed.

**Conclusions and clinical relevance:**

In summary, rats under ketamine-xylazine anaesthesia are susceptible to hypoxia. This may lead to increased delayed mortality related to hypoxia induced lung failure. Apparently, this is an underestimated problem. We highly recommend using additional oxygen insufflation in spontaneously breathing rats under ketamine-xylazine anaesthesia with basic monitoring such as measurement of oxygen saturation.

## Introduction

A new experimental rat model investigating the impact of liver cirrhosis on the healing of vascular damages was employed in our research group. Establishing new surgical animal models requires the selection of appropriate anaesthetic procedures. Selection criteria are the well-being of the trial animal, possible research interference, as well as financial and practical considerations such as ease of use or personal experience and preference [1, 2].

In general, intraperitoneal (IP) or inhalational anaesthetic induction is used in rats, as intravenous and intramuscular induction are inferior because of increased technical difficulty or painfulness [2]. In our experimental setting, we chose IP application, mainly because we planned to investigate the impact of liver cirrhosis on the heart, and inhalational anaesthetics such as fluranes are known to be cardioprotective [3–5]. In addition, the simplicity of IP administration was favourable.

With regard to the choice of drugs, it has to be considered that nearly every general anaesthetic has dose-dependent side effects. Therefore, most of the time, combinations of drugs are chosen to reduce side effects [2]. This is crucial for the refinement of animal trials according to the 3R rule of animal welfare (replacement, reduction, and refinement) postulated by Russel and Burch (1959). We chose a combination of ketamine and xylazine (KX), which is a common and well-described combination for rodents [6]. This combination seemed reasonable, as it reduces side effects. For instance, xylazine is known to induce hypotension, bradycardia, and respiratory depression [7, 8], whereas ketamine hydrochloride stimulates cardiopulmonary function in anaesthetic doses and has a wide safety margin [9]. Furthermore, great disadvantages of ketamine such as unstable analgesic effects and increased muscular tone are counteracted by xylazine [7, 9]. Only a few adverse events for this drug combination are described: a partially inconsistent analgesic effect, slight hypotension and hypoventilation, and in 3.3% of cases apnoea [7, 9, 10]. Furthermore, there are only a few reports of alarming fallouts, described as, for example, pulmonary oedema provoked by high doses of xylazine (21 mg kg^–1^ body weight [BW]) in young animals or high doses of ketamine (125 mg kg^–1^ BW with 10 mg kg^–1^ BW xylazine) [11–13].

Rats in our experiments were left spontaneously breathing, without intubation and without accessory oxygen as described several times for KX narcosis [14–16]. We observed unsustainable high mortality rates during the first 7 postoperative days but not during or immediately after surgery. Low survival rate in combination with the slightly reduced intraoperative saturation, the pathological findings of the lungs of decreased rats, and the knowledge that narcotics are able to reduce the breathing depth and that the intraoperative supine position can favour atelectasis, led us to assume a hypoxic genesis. This made us change the protocol by adding oxygen insufflation via nose cone during surgery.

The aim of the present study was to analyse the impact of oxygen supply during KX anaesthesia on the survival rate, haemodynamics, and animal condition after bile duct ligation, sham operation or dilatation of the carotid artery. For this purpose, two experimental groups were compared: animals with oxygen insufflation during surgery and those without (n = 12 in each intervention).

## Materials and methods

### Animals

Twenty healthy, male Sprague-Dawley rats from Janvier (RjHan:SD; Janvier Labs, Le Genest Saint Isle, France), weighing between 440 and 550 g preoperatively, were included in this study. Male rats were chosen, because only males reach the required size to use 2F balloon catheters (Edwards Lifesciences, Irvine, USA, 120602F) in the carotid artery [17]. All animals were housed under specific pathogen-free conditions, with a 12-hour light-dark cycle, in a temperature- (22 °C) and humidity-controlled environment (55% relative humidity), in rat filter top cages (Type 2000, Tecniplast, Hohenpreisenberg, Germany), and preferably in groups of two or three. Cages were changed twice a week. The rats were kept for at least 7 days prior to any procedure and were provided standard pellets for laboratory rats (Ssniff GmbH, Soest, Germany) and sterile, acidified water ad libitum.

### Experimental procedures

All experimental protocols in this study were reviewed and approved by the governmental animal care and use office (No 84–02.04.2016.A391, Landesamt für Natur-, Umwelt- und Verbraucherschutz Nordrhein-Westfalen, Recklinghausen, Germany) and all efforts were made to minimize animal suffering. The procedures were developed in accordance with *The National Research Council Guide for the Care and Use of Laboratory Animals*. They were originally designed and conducted to investigate the influence of liver cirrhosis on vascular healing after vessel damage in rats. For this purpose, two consecutive surgeries per animal were performed: first, a bile duct ligation to induce liver cirrhosis or an appropriate sham operation, and 4 weeks later, a balloon dilatation of the left carotid artery to induce vascular damage in both groups. Allocation to the bile duct or the sham group was ensured using an unpredictable random sequence.

After the first randomly assigned 12 interventions (in 8 animals), without additional oxygen supply, the protocol was amended because of high postoperative mortality rates. The initial 12 procedures were retrospectively summarised to the ambient air (AA) group (Table 1). The amended protocol added 2 litres of oxygen insufflation per minute via nose cone (HSE Anaesthesia Mask, 73–4861, Harvard Apparatus GmbH, Hugstetten, Germany) during surgery for the following 12 interventions (in 12 animals; O_2_ group). Thus, in total, 24 consecutive interventions with 28 days of follow-up in 20 male rats were included. We retrospectively compared the outcome of the described two groups (AA group vs. O_2_ group).

**Table 1:** Overview of the number of rats and the number of interventions included in the two study groups.

		1^st^ surgery		2^nd^ surgery
	Number of animals	Bile duct ligation	Sham operation	Balloon dilatation of the left carotid artery
**AA group**	8	5	3	4
**O**_**2**_ **group**	12	6	6	0

The ambient air (AA) group contained 8 animals which underwent a total of 5 bile duct ligations, 3 sham operations and 4 balloon dilatations of the left carotid artery. In the oxygen (O_2_) group, a total of 12 rats with 6 bile duct ligations and 6 sham operations were included.

Overall, 11 bile duct ligations, nine sham operations with a median laparotomy [18], and four balloon dilatations of the left carotid artery with a median incision in the ventral neck [19] were included in this study. The exact distribution between the two groups can be found in Table 1. All experiments were performed between 8:00 am and 4:00 pm in the same laboratory of the animal facility.

### Anaesthesia

All rats were anaesthetised with an IP injection of 60 mg kg^–1^ BW (S)-ketamine (Ketanest S, 25 mg mL^–1^, Pfizer, New York, USA) and 10 mg kg^–1^ BW xylazine (Proxylaz 2%, Prodivet Pharmaceuticals S.A., Raeren, Belgium). During the first eight interventions (these are all animals from the AA group), rats additionally received 0.01 to 0.03 mg kg^–1^ BW buprenorphine IP. This was later omitted because of the high mortality rate and no observed signs of pain. All animals were allowed to breathe spontaneously, without intubation. The first 12 operations were carried out under room air conditions (AA group), whereas oxygen insufflation with a flow of 2 L min^–1^ via a nose cone was added to the protocol for the last 12 procedures. Eye ointment (Bepanthen^R^, Bayer, Leverkusen, Germany) prevented eye damage during unconsciousness. Basic monitoring included electrocardiography (ECG) via needle electrodes and measurement of blood oxygen saturation via pulse oximetry at the paw (Masimo Radical 7 Blue Screen, Irvine, USA). Monitoring was connected immediately after anaesthetic induction and was displayed and recorded continuously for subsequent analysis (Powerlab, LabChart, ADInstruments, Dunedin, New Zealand). Adequate depth of anaesthesia was periodically verified by the absence of a nociceptive response to tail tip and interdigital pinch. Such a nociceptive response could either be any reactive movement or reflex (usually a pedal withdrawal reflex) or a noticeable raise in heart or breathing rate. In case of inadequate analgesia, an additional administration of 25 mg kg^–1^ BW (S)-ketamine IP followed.

Heat management was conducted with a rectal temperature probe and a feedback-controlled heating pad, analogue to Roehl et al., to prevent overheating or hypothermia during anaesthesia (TCAT-2LV controller, Physitemp, Clifton, USA) [20].

According to the guidelines of the Gesellschaft für Versuchstierkunde–the Society for Laboratory Animal Science (GV-SOLAS), postoperative analgesia was guaranteed by means of subcutaneously (SC) applied metamizole (100 mg kg^–1^ BW, diluted to 100 mg mL^–1^) (novaminsulfon ratio 1 g/2 mL; Ratiopharm, Ulm, Germany) [21], and a preemptive local infiltration of ropivacaine in the wound margin (25 mg kg^–1^ BW (Ropivacain Kabi 1% [10 mg mL^–1^], Fresenius) was administered to minimise pain.

Atipamezole hydrochloride (2 mg kg^–1^ BW, IP) (Antisedan^R^, 5 mg mL^–1^, Orion Pharma, Espoo, Finland) was used in all rats as an antidote of xylazine, once surgery was completed [22].

### Follow-up

Postoperative observation was carried out in a small-animal intensive care unit (Vetario, Weston-super-Mare, UK) until full recovery. The unit provided a controlled temperature environment in both groups and additionally supplied elevated oxygen levels in the O_2_ group by introducing 2 litres of oxygen per minute. On the following days, the rats were examined, scored, and weighed at least daily. The score sheet, used to evaluate the animals’ discomfort, included four factors: BW, general condition, spontaneous behaviour, and model-specific clinical criteria (such as signs of bleeding, stroke, abdominal tension, or encephalopathy). Each of the four factors could reach a maximum score of 20 points. The score of the single factors were added to a total score. A total score of 20 points was considered as severe stress and required the consultation of the animal welfare officer, the introduction of veterinary treatment and the euthanasia of the animal, if deemed necessary. If euthanasia was required, it was performed under deep anaesthesia by exsanguination in combination with organ retrieval (heart). An additional injection of metamizole (100 mg kg^–1^ BW, diluted to 100 mg mL^–1^, SC) was provided if there was any indication of persistent postoperative pain. The observation period for this study was 28 postoperative days to reliably display perioperative complications.

### Tissue sampling

All deceased animals were autopsied for macroscopic hints concerning the cause of death. Lung, heart, and liver tissues were taken from selected animals. The tissues were fixed in 4% formalin, dehydrated through graded alcohol series (xylene, 100%, 96% and 70% alcohol and distilled water), and embedded in paraffin wax for histological analysis. Paraffined tissues were cut in sections of 3 μm. Sections were strained with haematoxylin and eosin (Merck KGaA, Darmstadt, Germany, 109249 and 117081) according to the following scheme: 3 minutes covered with haematoxylin, 5 minutes watering in fluent normal water, 1 minute covered with eosin, tapped into water, pulled through an ascending alcohol series and covered with Roti^R^-Histokitt (Carl Roth GmbH & Co. KG, Karlsruhe, Germany, 6638.1). Subsequently, the histological sections were examined under light microscopy with a LEICA DM 2500 microscope (Leica Microsystems, Nussloch, Germany). Images were captured with a LEICA DFC420 C camera (Leica Microsystems) under bright field illumination at 40x and 100x magnification. The stained sections were used for the descriptive assessment of histological changes.

### Hemodynamic and respiratory data analysis

All parameters were calculated from continuous recordings by 1-minute averages every 10 minutes for HR, SpO_2_, and respiratory rate (RR). RR was determined by respiratory-induced changes of R-wave amplitudes, as described earlier [23].

### Statistics

Statistical analyses were performed with SPSS^R^ Statistics Version 24 (IBM Corporation, Armonk, USA), and data were plotted by means of GraphPad PRISM 8 (GraphPad Software, San Diego, USA). A *p*-value <0.05 was considered statistically significant. The Mantel-Cox test was used to describe the effect of oxygen supply, type of intervention (bile duct ligation, sham operation, or carotid dilatation), and the use of buprenorphine on survival. A Chi-square test was used to determine whether additional ketamine injections differed between the groups and if the number of these injections correlated with survival. A generalized linear model was carried out for analysis of SpO_2_ values, HR, RR, BW, and score values.

All results are reported as mean ± standard error of the mean.

## Results

**Fig 1:**
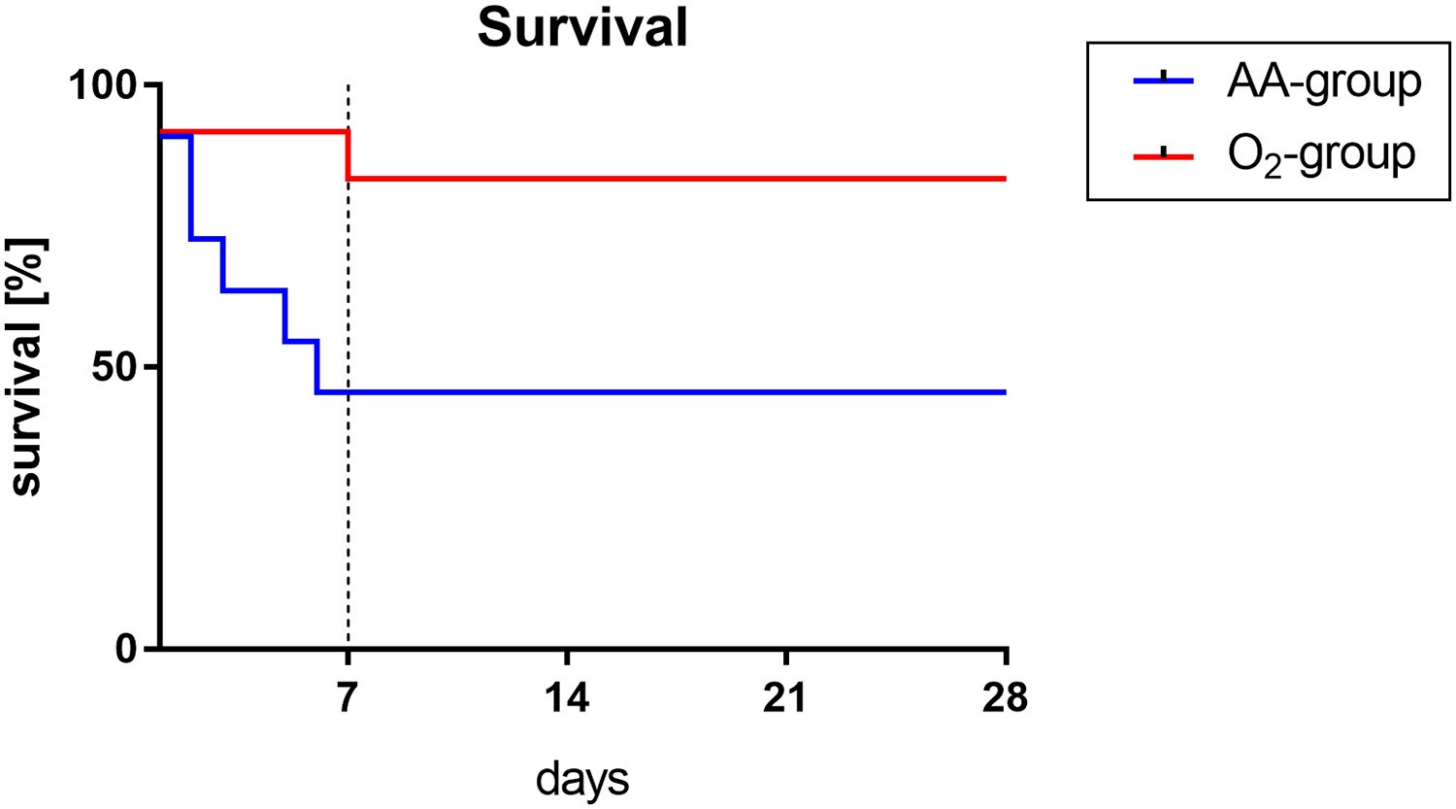
28-day survival of rats after KX anaesthesia with (O_2_ group) or without (AA group) additional oxygen insufflation. The survival curve (Kaplan-Meier analysis) revealed that the survival rate during the follow-up period of 28 days was significantly higher in rats who received additional oxygen insufflation intra- and postoperatively (O_2_ group) compared to rats who only breathed ambient air peri- and intraoperatively (AA group) (p = 0.036).

A significant lower mortality rate in the O_2_ group compared with the AA group could be observed (*p* = 0.036) during the first seven days after KX anaesthesia: survival in the AA group was only 42% (5/12) compared with 83% (10/12) in the O_2_ group (Fig 1).

The type of intervention (bile duct ligation, sham operation, or carotid dilatation) did not affect the outcome significantly (*p* = 0.091): in the AA group (vs. the O_2_ group), four out of five (vs. one out of six) rats died following bile duct ligation, zero out of three (vs. one out of six) after sham operation, and three out of four subsequent to balloon dilatation of the left carotid artery.

Administration of buprenorphine did not influence the survival rate in the AA group: 50% (4/8) with buprenorphine versus 25% (1/4) without buprenorphine (*p* = 0.549). No animal in the O2 group received buprenorphine.

Overall, in the O_2_ group, seven animals needed collectively 15 boluses of additional ketamine, whereas in the AA group, only three animals received together five boluses of supplemental ketamine (in each case, 25 mg kg^−^1 BW IP). Whether an animal needed additional ketamine had no significant effect on survival: survival rate was 64% (9/14) of rats that needed no supplemental ketamine injections, 33% (1/3) of those that needed one, 75% (3/4) of those that needed two, and 67% (2/3) of those that needed three injections (*p* = 0.706). In addition, group membership had no significant influence on the number of ketamine injections (*p* = 0.121). Additional postoperative metamizole was needed five times (in four animals) in the O_2_ group only.

**Fig 2:**
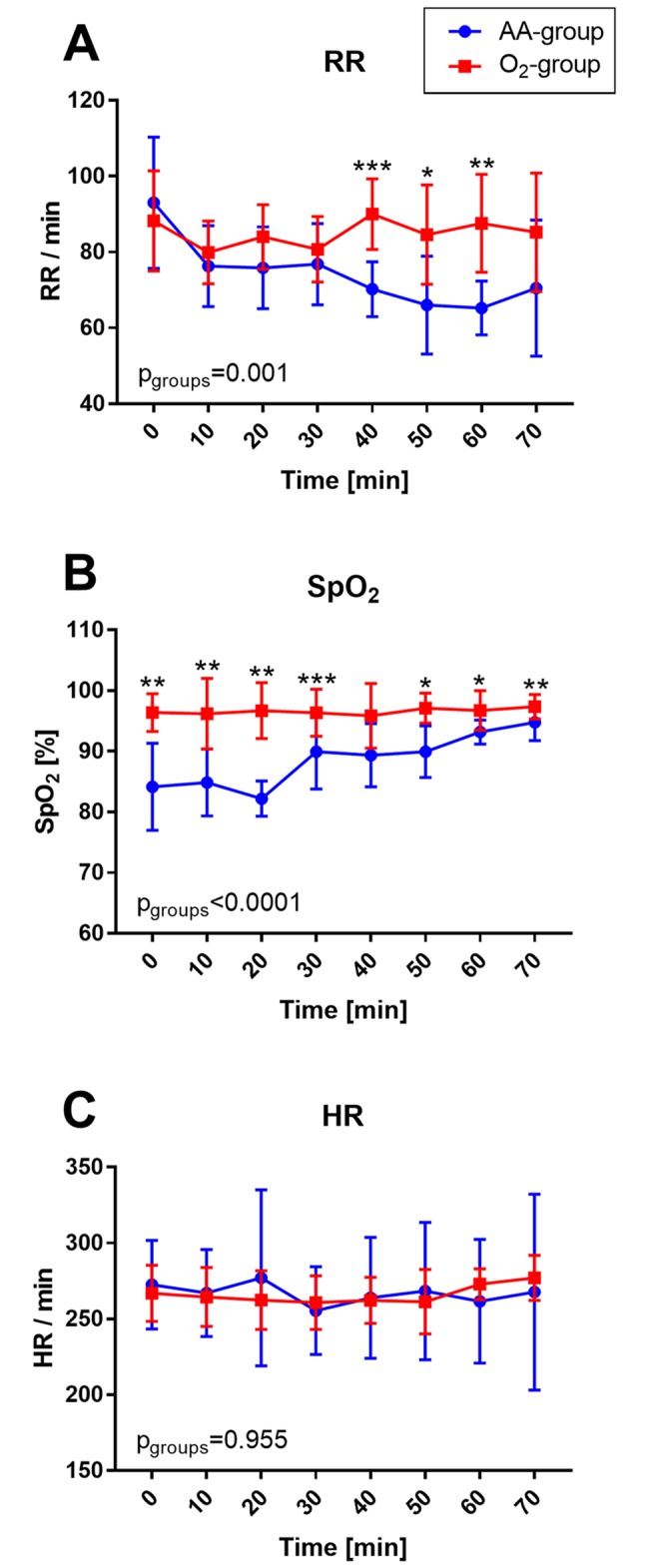
Intraoperative time course of (A) respiratory rate (RR), (B) oxygen saturation (SpO_2_) and (C) heart rate (HR) of male Sprague Dawley rats under KX anaesthesia depending on the additional oxygen supply. Rats in the ambient air (AA) group only breathed ambient air whereas rats in the oxygen (O_2_) group had an increased oxygen supply intra- and postoperatively. The graphs show significantly increased saturation values in the O_2_ group and significantly higher respiratory rates after 40 minutes of anaesthesia compared to the AA group. No group differences are visible in heart rate. Symbols mark significant differences between groups at the time points (*<0.05; **<0.005; ***<0.0005; ****<0.0001).

Intraoperative SpO_2_ values in all animals averaged 94.08 ± 0.59%, with significant differences between the groups (88.55 ± 4.44% [AA] vs. 96.59 ± 0.49% [O_2_]) (*p* < 0.0001). The SpO_2_ curve showed initially reduced values in the AA group that converged to the higher levels of the O_2_ group over time (Fig 2B). Apart from the SpO_2_ values 40 minutes after anaesthetic induction, the SpO_2_ values differed significantly between the two groups over the entire intraoperative observation period of 70 minutes. The SpO_2_ values in the AA group were similar to the data of Giroux et al., whose animals partially showed lung oedema and pulmonary effusion [13].

In addition, retrospective analysis of the breathing rate exposed a respiratory depression that was predominant in the AA group (Fig 2A). Significant differences in breathing frequency between the groups occurred 40 to 60 minutes after anaesthetic induction (*p* < 0.05). In contrast, intraoperative HR did not vary significantly between the groups (*p* = 0.955) and remained stable over time (Fig 2C). HR averaged 267 ± 7 beats per minute (bpm) (267 ± 7 bpm [AA] vs. 266 ± 6 bpm [O_2_]).

**Fig 3:**
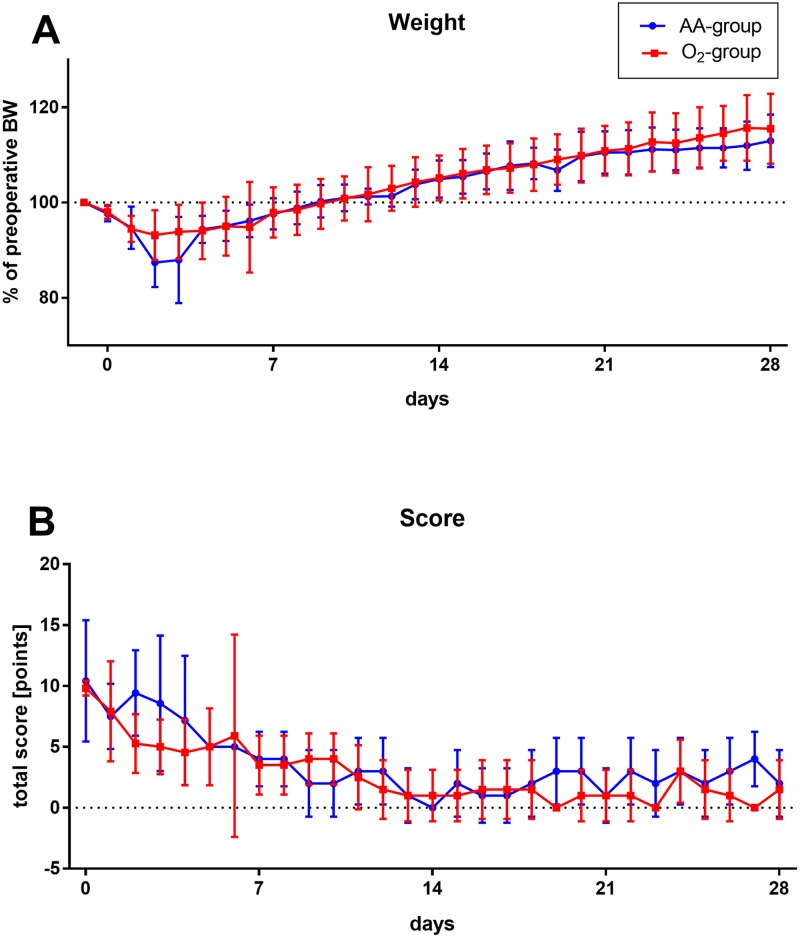
Postoperative weight and score development of male rats during the follow-up period of 28 days after KX anaesthesia with (O_2_ group) or without (AA group) additional intra- and postoperative oxygen insufflation. Course of (A) postoperative, in relation to preoperative body weight (BW) and (B) the postoperative score values illustrated for ambient air (AA) and oxygen (O_2_) group. A score value of altogether 5 to 9 points was defined as low, 10 to 19 points as medium and equal or greater than 20 points as high distress. There are no significant group differences in weight (*p* = 0.521) or score values (*p* = 0.259). However, the average weight in rats that only received ambient air tends to be slightly lower on the second and third postoperative day and the score value from the second to the fourth day slightly higher than in animals with additional oxygen.

Preoperative BW was comparable between the groups (487 ± 10 g versus 488 ± 10 g). Postoperative average weight loss was slightly more pronounced in the group without oxygen supply after 2 and 3 days without reaching statistical significance (*p* = 0.521; Fig 3A). Weight recovery was reached about 9 days after surgery in both groups. The determined stress score values were not significantly different (*p* = 0.259; Fig 3B).

**Fig 4:**
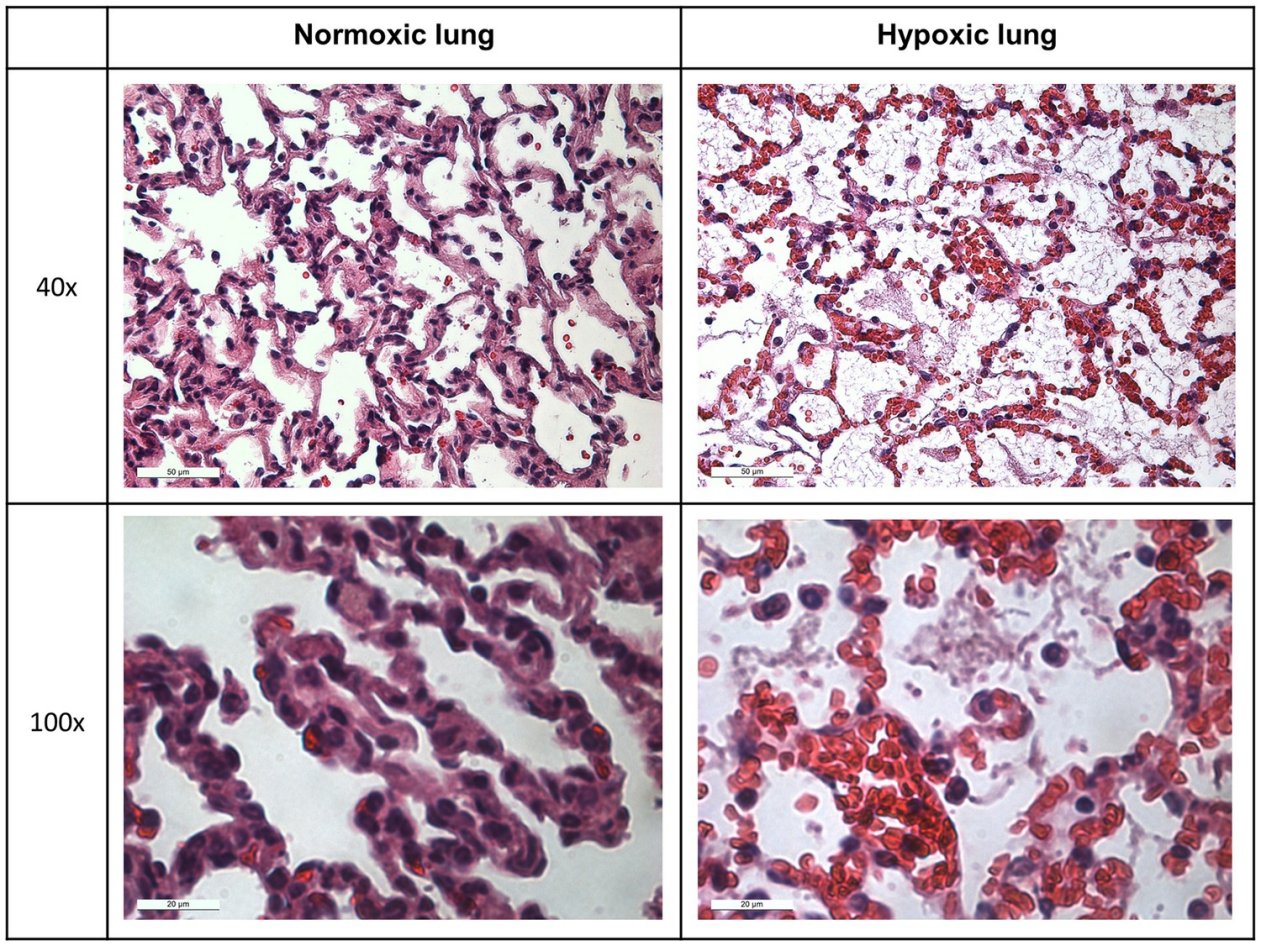
Exemplary histological images of lung tissue from both hypoxic and normoxic rats in 40x and 100x magnifications. The images of the hypoxic lung show intra-alveolar fibrin depositions and increased interstitial erythrocytes. Both changes indicate increased vascular permeability in hypoxic rats.

Autopsies revealed conspicuous, red lungs with signs of pulmonary oedema in six of seven cases in the AA group (86%) and only one of two cases in the O_2_ group (50%). Exemplary histological analysis of lungs out of the AA group showed numerous interstitial erythrocytes (Fig 4), consistent with the macroscopic red picture of the lung. In addition, intra-alveolar fibrin deposits were apparent in these lungs (Fig 4). Both indicate an increased vascular permeability.

In contrast, only one animal in each group died for reasons other than pulmonary issues, most likely because of proceeded cirrhosis of the liver following bile duct ligation.

## Discussion

The motivation for this study arose as we observed unexpected and intolerable high mortality rates in male Sprague-Dawley rats following KX anaesthesia who were breathing AA (42% survival) and a remarkable improvement due to oxygen supply (83% survival). The main cause of death seemed to be related to hypoxia-induced lung injury, as suggested by pathological findings.

In light of the high initial mortality rate and observed lung damage, our first suspicion was that the opioid buprenorphine in combination with KX was causing excessive respiratory depression during and immediately after surgery and thereby induced hypoxaemia. This hypothesis was supported by the findings of Roughan et al., who described a mortality of 56% (9/16 rats) with increased respiratory depression and postoperative pulmonary oedema with the combination of buprenorphine and ketamine/medetomidine [24]. However, contrary to our expectations, administration of buprenorphine did not influence the mortality rate in our experiments. Consequently, KX narcosis seemed to be the decisive factor leading to a lung damage through respiratory depression. Hypoxaemia is known, in principle, to cause lung damage in rats [25]. This damage starts 8 hours after the hypoxic incident with pulmonary oedema, most likely due to hypoxic pulmonary vasoconstriction, sympathetic activation, and increased vascular permeability, and is followed by inflammation, pulmonary fibrosis, and vascular hypertrophy [25]. The mechanistic explanation suits the observed pulmonary oedemas after KX anaesthesia seen in this experiment.

The high mortality rate occurred although spontaneous breathing per se without intubation or supplemental oxygen is unproblematic in rats under KX anaesthesia according to the literature; but in most cases describing KX anaesthesia, no statement is provided as to whether additional oxygen was administered [14–16, 26–28]. Therefore, it must be assumed that oxygen is not commonly supplied during KX narcosis. Supplemental oxygen provided via nose cone or mask is reported in only few other papers [29, 30], sometimes during only anaesthetic induction [7]. To the best of our knowledge, only few papers mention the importance of oxygen, which matches our findings: Ford et al. stated that it is important to monitor vital parameters during anaesthesia because, especially for injectable anaesthetics, hypoxia is specified as a threat [15]. The authors used oxygen inhalation through a nose cone to prevent hypoxia. Nevertheless, they did not demonstrate the fatal consequences that omission of additional oxygen might entail, especially the delayed deaths after up to 7 days. In line with these findings, Lee and Jones were able to detect hypoxemia with fluctuating respiratory frequencies in mice under KX anaesthesia [31]. By supplementation of oxygen (via a tube and funnel) they were able to demonstrate that hypoxemia was effectively avoided, respiratory rate and physiological condition were stabilized and survival of mice under KX anaesthesia was significantly prolonged.

In contrast to our findings and these descriptions, oxygen is sometimes termed only to enhance laboratory values, such as partial pressure and SpO_2_, and no influence on survival is described. On the contrary, for ventilated, not spontaneously breathing, rats or during volatile anaesthesia, the oxygen supply is often described in more detail, probably as volatile anaesthetics are mostly supplied using oxygen as a carrier medium.

Our results show the importance of oxygen supply during KX anaesthesia, as hypoxia leads to increased mortality rates not only during surgery but also up to seven days later. Because of the delayed incidence, a correct classification of death as late complication of anaesthesia is often not obvious. We suspect it might be mistakenly attributed to the experimental rat model, especially in the case of new or possibly stressful experimental designs.

Anaesthesia with KX is known to trigger respiratory depression and a decrease in SpO_2_ in rats [7, 32, 33], as it did in this experiment. The initial drop of SpO_2_ in the AA group is not shown here, as the period in which the sedation starts working is not recorded, in order to minimise stress for the animals. The observed breathing frequencies under KX anaesthesia, as well as their course over time, were consistent with previous findings [16, 32, 33]. Only the drop in frequency was comparatively delayed in our observations: we observed minimal RRs after 50 minutes, whereas Dodelet-Devillers et al. and Wellington et al. already observed comparative values after 25 to 30 minutes [16, 33]. Interestingly and counterintuitively, respiratory depression occurred predominantly in the AA group and additional oxygen increased RR. One would expect hypoxia in combination with hypercapnia to raise the breathing rate, as was the case in the study by Jimenez-Ruiz et al. [32]. However, blood gas analysis was not included in the experimental design; therefore, arterial O_2_ and CO_2_ values cannot be given to further illuminate this observation.

To avoid complications such as hypoxia, the appropriate choice of monitoring during anaesthesia of rats has to be considered. For example, there are different possibilities to monitor the breathing rate: either by means of the ECG or SpO_2_ curve, by impedance measurement, or by an acceleration sensor. We received good values using the ECG amplitude. However, this does not provide any information about the efficiency of breaths (the breathing depth), which is even more difficult to monitor.

In addition, continuous monitoring of the temperature as well as appropriate heat supply during anaesthesia is essential for the survival of the animals [20].

Missing blood pressure measurements are certainly a limitation in our experimental design, as KX anaesthesia as well as hypoxia is known to reduce both systolic and diastolic blood pressure [8, 14]. The distinct reduction continues even beyond the duration of the sedation and should not be overlooked, as hypotension can cause lethal consequences as well [14]. Furthermore, inconsistent chronotropic reactions of the heart to hypoxia are reported: some authors observed reactive tachycardia possibly though sympathetic activation, whereas others detected no chronotropic reaction or even a clear reduction of the frequency [8, 25]. We could not observe any differences in HR in the rats with reduced SpO_2_ and possible hypoxia. Consequently, there was no correlation between outcome and HR of the animals. This could also be due to the α2-agonist activity of xylazine and thereby the drug-induced sympathicolysis with bradycardia in both groups. According to the literature, this effect is described for xylazine alone and in combination with ketamine [8, 10, 14]. A feasible reason why we did not detect any initial frequency drop triggered by KX anaesthesia could be the start of our recording period only after induction of anaesthesia.

It is highly unlikely that the reason for the higher mortality in the AA group is caused by the depth of anaesthesia, as both groups, the AA and O_2_ group, initially received exactly the same BW-adjusted dose of anaesthetics and, overall, the animals in the O_2_ group received more subsequent injections of ketamine. Since all animals in both groups also received the antidote atipamezole, resedation due to a weaning effect of the antidote can also be ruled out as a reason for the differences between the groups. HR is a regularly used indicator for the depth of anaesthesia and for the cardiac condition of the animal. Average HRs identified in this study (267 ± 7 bpm) are similar to those described by Picollo et al. under KX anaesthesia [14]. But as discussed above, because of damped cardiac responsiveness on the basis of xylazine, we believe HR alone is an insufficient key indicator for monitoring the depth of anaesthesia and general condition of the rat under KX anaesthesia.

All animals were kept specific-pathogen free, and no signs of infection could be observed, so pathogen influences as a cause of death are unlikely, especially as oxygen supply entailed the changes in survival.

Overall, exact planning and optimal setup of anaesthetic protocols is necessary both to reduce the numbers of required animals and to avoid interactions with primary research objectives.

The study has all the shortcomings of a retrospective study with small cohorts. In particular, the unequal distribution of operations in the two groups limits the validity of the study. However, although the cohorts are very small, it must be noted that the extremely low survival of the AA group is nevertheless reportable and a further increase in the number of cases seems unacceptable for ethical reasons.

## Conclusions

In summary, our data suggest that rats under KX anaesthesia, that are spontaneously breathing AA, are at risk of fatal pulmonary complications. The occurrence of such complications can be significantly reduced with the aid of an adequate combined intra- and postoperative oxygen supply (e.g., via nose cone and a small-animal intensive care unit) in a suitable monitoring setting. The latter includes at minimum the measurement of intraoperative SpO_2_ and RR. This seemingly trivial aspect appears to be disregarded in many research models using KX anaesthesia in rats, even though this simple and straightforward modification could lead to safer and more reliable anaesthesia in rats. More research focusing on the exact effect in a prospective manner could further increase the insight into rat narcosis management especially for KX anaesthesia. In addition, accurate reports on complications are necessary, even if they do not appear to be relevant at first glance.

## Supporting information

S1 File The values behind the means, standard errors and graphs. (SAV)

S2 File ARRIVE (Animal Research: Reporting of In Vivo Experiments). Filled ARRIVE guidelines checklist.
(PDF)
